# Supplementary material for: Is recovery just the beginning? Persistent symptoms and health and performance deterioration in post-COVID-19, non-hospitalized university students—a cross-sectional study
Source: Biol Methods Protoc. 2023 Dec 6;8(1):bpad037. doi: 10.1093/biomethods/bpad037 (PMC10739555; doi:10.1093/biomethods/bpad037)
Supplement: bpad037_Supplementary_Data [file bpad037_supplementary_data.docx]

# Supplementary Materials

**Supplementary Table S1 – Health & cognitive assessment indices and variables**

| **No.** | **Variable** | **Description** |
| --- | --- | --- |
| **Indices** | | |
| 1 | Physical sickness score | Mean of Z-scores of 19 variables related to physical health (19-37) measured on a 6-point ordinal scale |
| 2 | Mental sickness score | Mean of Z-scores of six variables related to mental health (38-44) measured on a 6-point scale |
| 3 | Fatigue score | Mean of Z-scores of five variables related to fatigue measured on a 6-point scale |
| 4 | Error rate score | Mean of inverted Z-scores from variables 6-8, 11, and 13 |
| 5 | Reaction time score | Mean of Z-scores of variables 12, 14, 18 |
| **Source variables** | | |
| ***Cognitive performance*** | | |
| 6 | Evolution test result | Number of correct answers in the Evolutionary Biology exam, with a maximum of 30 |
| 7 | Intelligence | Number of correct answers in the Cattel 16PF test (Variant A, Scale B), with a maximum of 12 |
| 8 | Cognitive reflection test | Number of correct answers in the CRT, with a maximum of 3 |
| 9 | Recognition memory test | Number of correct answers in the Meili recognition memory test, with a maximum of 12 |
| 10 | Free recall memory test | Number of correct answers in the Meili free memory test, with a maximum of 12 |
| 11 | Choice test accuracy | Number of correct answers in the Choice test, with a maximum of 6 |
| 12 | Choice test reaction time | Mean reaction time in all six trials of the Choice test (in ms) |
| 13 | Stroop test accuracy | Number of correct responses across all 15 trials in the Stroop Test, with a maximum of 15 |
| 14 | Stroop test reaction time | Mean reaction time in all 15 trials in the Stroop Test (in ms) |
| 15 | Stroop test reaction time 1^st^ part | Mean reaction time in trials 1-5 of the Choice test (in ms) |
| 16 | Stroop test reaction time 2^nd^ part | Mean reaction time in trials 6-10 of the Choice test (in ms) |
| 17 | Stroop test reaction time 3^rd^ part | Mean reaction time in trials 11-15 of the Choice test (in ms) |
| 18 | Reading time | Mean of Z-scores of the time taken to read the instructions for all included tests |
| ***Physical health issues*** | | |
| 19 | Allergies | Rated using 6-point ordinal scales anchored by 'never' and 'daily or more frequently' |
| 20 | Skin disorders | Rated using 6-point ordinal scales anchored by 'never' and 'daily or more frequently' |
| 21 | Digestive tract disorders | Rated using 6-point ordinal scales anchored by 'never' and 'daily or more frequently' |
| 22 | Metabolic disorders | Rated using 4-point ordinal scales anchored by 'definitively no' and 'definitively yes' |
| 23 | Common infectious diseases | Rated using 5-point ordinal scales anchored by 'never' and 'two or more times a week' |
| 24 | Orthopedic disorders | Rated using 6-point ordinal scales anchored by 'never' and 'daily or more frequently' |
| 25 | Neurological disorders | Rated using 6-point ordinal scales anchored by 'never' and 'daily or more frequently' |
| 26 | Headaches | Rated using 6-point ordinal scales anchored by 'never' and 'daily or more frequently' |
| 27 | Physical pains | Rated using 6-point ordinal scales anchored by 'never' and 'daily or more frequently' |
| 28 | Chronic physical problems | Rated using 6-point ordinal scales anchored by 'never' and 'daily or more frequently' |
| 29 | Antibiotics in the last year | Rated using 6-point ordinal scales anchored by 0. 'never' and 6 'more than 5 times' |
| 30 | Antibiotics in the last 3 years | Rated using 7-point ordinal scales anchored by 0. 'never' and 6 'more than 5 times' |
| 31 | Doctor visits | Rated using 5-point ordinal scales anchored by 0. 'never' and 'two or more times a week' |
| 32 | Hospital visits in the past 5 years | Rated using 6-point ordinal scales anchored by 0. 'never' and 6 'more than 5 times' |
| 33 | Prescribed drugs for physical health | Rated using 7-point ordinal scales anchored by 0. 'never' and 6 'more than 5 times' |
| 34 | Physical health disparity | Rated using 6-point ordinal scales anchored by 'definitively no' and 'definitively yes' |
| 35 | Feeling physically unwell today | Rated using 6-point ordinal scales anchored by 'definitively no' and 'definitively yes' |
| 36 | Feeling physically unwell usually | Rated using 6-point ordinal scales anchored by 'definitively no' and 'definitively yes' |
| 37 | Expected shorter lifespan | Rated using 6 response options ranging from 'more than 99 years' to 'less than 60 years' |
| ***Mental health issues*** | | |
| 38 | Depression | Rated using 6-point ordinal scales anchored by 'never' and 'daily or more frequently' |
| 39 | Anxiety | Rated using 6-point ordinal scales anchored by 'never' and 'daily or more frequently' |
| 40 | Phobia | Rated using 6-point ordinal scales anchored by 'never' and 'daily or more frequently' |
| 41 | Obsession | Rated using 6-point ordinal scales anchored by 'never' and 'daily or more frequently' |
| 42 | Other mental health problems | Rated using 6-point ordinal scales anchored by 'never' and 'daily or more frequently' |
| 43 | Prescribed drugs for mental health | Rated using 7-point ordinal scales anchored by 0. 'never' and 6 'more than 5 times' |
| 44 | Mental health disparity | Rated using 6-point ordinal scales anchored by 'definitively no' and 'definitively yes' |
| 45 | Feeling mentally unwell today | Rated using 6-point ordinal scales anchored by 'definitively no' and 'definitively yes' |
| 46 | Feeling mentally unwell usually | Rated using 6-point ordinal scales anchored by 'definitively no' and 'definitively yes' |
| ***Fatigue*** | | |
| 47 | Tired usually | Rated using 6-point ordinal scales anchored by 'definitively no' and 'definitively yes' |
| 48 | Tired now | Rated using 6-point ordinal scales anchored by 'definitively no' and 'definitively yes' |
| 49 | Tired after work | Rated using 6-point ordinal scales anchored by 'never' and 'always' |
| 50 | Feeling tired after bus travel | Rated using 6-point ordinal scales anchored by 'never' and 'always' |
| 51 | Feeling tired after train travel | Rated using 6-point ordinal scales anchored by never' and 'always' |

**Supplementary Table S2 – Descriptive statistics for the dependent variables**

|  | Sex | | | | | COVID-19 | | | | |
| --- | --- | --- | --- | --- | --- | --- | --- | --- | --- | --- |
|  | Mean | | SD | | Cohen d | Mean | | SD | | Cohen d |
|  | women | men | women | men |  | No | Yes | No | Yes |  |
| Physical sickness score | 0.08 | -0.10 | 0.52 | 0.48 | -0.35 | -0.04 | 0.04 | 0.57 | 0.49 | 0.15 |
| Mental sickness score | 0.09 | -0.16 | 0.70 | 0.61 | -0.37 | -0.02 | 0.02 | 0.60 | 0.71 | 0.05 |
| Fatigue score | 0.06 | -0.13 | 0.75 | 0.73 | -0.25 | -0.15 | 0.06 | 0.64 | 0.78 | 0.29 |
| Error rate score | -0.02 | 0.08 | 0.51 | 0.53 | 0.19 | 0.01 | 0.01 | 0.51 | 0.53 | 0.01 |
| Reaction time score | 0.03 | -0.07 | 0.63 | 0.65 | -0.16 | -0.10 | 0.04 | 0.50 | 0.68 | 0.23 |
|  | Source variables | | | | | | | | | |
| Evolutionary biology test result | 21.79 | 21.32 | 3.49 | 3.12 | -0.14 | 21.32 | 21.76 | 3.56 | 3.30 | 0.13 |
| Intelligence | 9.59 | 10.22 | 1.48 | 1.27 | 0.45 | 9.69 | 9.85 | 1.52 | 1.41 | 0.11 |
| Cognitive reflection test | 1.36 | 1.72 | 1.15 | 1.02 | 0.33 | 1.68 | 1.39 | 1.15 | 1.10 | -0.26 |
| Recognition memory test | 10.71 | 9.84 | 1.62 | 2.17 | -0.48 | 10.27 | 10.49 | 2.19 | 1.70 | 0.11 |
| Free memory test | 7.26 | 6.41 | 2.86 | 2.95 | -0.29 | 6.86 | 7.03 | 2.92 | 2.91 | 0.06 |
| Choice test accuracy | 6.00 | 5.97 | 0.00 | 0.17 | -0.30 | 6.00 | 5.99 | 0.00 | 0.12 | -0.14 |
| Choice test reaction time | 0.06 | -0.12 | 0.60 | 0.83 | -0.27 | -0.06 | 0.03 | 0.63 | 0.71 | 0.13 |
| Stroop test accuracy | 14.05 | 13.76 | 1.72 | 1.72 | -0.17 | 14.02 | 13.93 | 1.59 | 1.78 | -0.05 |
| Stroop test reaction time | 1.11 | 1.08 | 0.47 | 0.40 | -0.08 | 1.05 | 1.12 | 0.29 | 0.50 | 0.17 |
| Stroop test reaction time 1 | 1.26 | 1.12 | 0.81 | 0.38 | -0.19 | 1.06 | 1.28 | 0.30 | 0.81 | 0.33 |
| Stroop test reaction time 2 | 0.85 | 0.90 | 0.40 | 0.55 | 0.11 | 0.93 | 0.84 | 0.42 | 0.47 | -0.19 |
| Stroop test reaction time 3 | 1.24 | 1.23 | 0.77 | 0.66 | 0.00 | 1.18 | 1.26 | 0.46 | 0.83 | 0.10 |
| Reading time | 0.02 | -0.04 | 0.38 | 0.34 | -0.15 | -0.08 | 0.03 | 0.35 | 0.37 | 0.30 |
| Allergies | 1.57 | 1.61 | 1.58 | 1.66 | 0.02 | 1.52 | 1.61 | 1.62 | 1.60 | 0.06 |
| Skin disorders | 1.36 | 1.45 | 1.47 | 1.67 | 0.06 | 1.41 | 1.38 | 1.64 | 1.50 | -0.02 |
| Digestive tract disorders | 1.41 | 1.49 | 1.19 | 1.19 | 0.07 | 1.48 | 1.42 | 1.30 | 1.13 | -0.05 |
| Metabolic disorders | 0.63 | 0.28 | 0.97 | 0.55 | -0.40 | 0.34 | 0.59 | 0.76 | 0.91 | 0.29 |
| Common infectious diseases | 1.49 | 1.36 | 0.61 | 0.60 | -0.22 | 1.38 | 1.48 | 0.55 | 0.63 | 0.16 |
| Orthopedic disorders | 2.04 | 1.57 | 1.40 | 1.25 | -0.35 | 1.78 | 1.94 | 1.36 | 1.37 | 0.11 |
| Neurological disorders | 0.76 | 0.63 | 1.36 | 1.17 | -0.10 | 0.58 | 0.78 | 1.05 | 1.39 | 0.16 |
| Headaches | 2.55 | 2.15 | 1.02 | 0.95 | -0.40 | 2.26 | 2.49 | 1.03 | 0.99 | 0.22 |
| Physical pains | 2.11 | 1.84 | 1.19 | 1.15 | -0.23 | 1.98 | 2.04 | 1.24 | 1.16 | 0.04 |
| Chronic physical problems | 1.36 | 1.04 | 1.47 | 1.21 | -0.23 | 1.03 | 1.36 | 1.28 | 1.44 | 0.23 |
| Antibiotics in the last year | 0.49 | 0.27 | 0.68 | 0.48 | -0.35 | 0.38 | 0.44 | 0.63 | 0.64 | 0.10 |
| Antibiotics in the last 3 years | 1.58 | 0.88 | 1.57 | 1.23 | -0.48 | 1.11 | 1.46 | 1.31 | 1.58 | 0.24 |
| Doctor visits | 1.18 | 1.09 | 0.63 | 0.71 | -0.14 | 1.20 | 1.13 | 0.72 | 0.63 | -0.12 |
| Hospital visits in the past 5 years | 0.23 | 0.09 | 0.58 | 0.29 | -0.28 | 0.20 | 0.17 | 0.48 | 0.52 | -0.06 |
| Prescribed drugs for physical health | 0.40 | 0.36 | 0.74 | 0.64 | -0.06 | 0.39 | 0.38 | 0.77 | 0.68 | -0.01 |
| Physical health disparity | 3.44 | 3.20 | 0.95 | 1.27 | -0.22 | 3.17 | 3.44 | 1.11 | 1.04 | 0.25 |
| Feeling physically unwell today | 3.93 | 3.64 | 1.26 | 1.37 | -0.23 | 3.63 | 3.94 | 1.32 | 1.29 | 0.24 |
| Feeling physically unwell usually | 3.88 | 3.57 | 1.11 | 1.09 | -0.28 | 3.67 | 3.82 | 1.06 | 1.14 | 0.14 |
| Expected shorter lifespan | 3.35 | 3.55 | 1.19 | 1.31 | 0.17 | 3.38 | 3.43 | 1.21 | 1.25 | 0.04 |
| Depression | 1.74 | 1.54 | 1.49 | 1.36 | -0.14 | 1.66 | 1.68 | 1.35 | 1.49 | 0.02 |
| Anxiety | 2.51 | 2.12 | 1.41 | 1.33 | -0.28 | 2.55 | 2.31 | 1.17 | 1.49 | -0.17 |
| Phobia | 1.79 | 1.31 | 1.42 | 1.28 | -0.35 | 1.70 | 1.60 | 1.42 | 1.38 | -0.07 |
| Obsession | 2.28 | 2.11 | 1.76 | 1.74 | -0.10 | 2.22 | 2.22 | 1.80 | 1.74 | 0.00 |
| Other mental health problems | 1.47 | 0.98 | 1.60 | 1.39 | -0.32 | 1.34 | 1.30 | 1.44 | 1.60 | -0.03 |
| Prescribed drugs for mental health | 0.22 | 0.07 | 0.69 | 0.26 | -0.24 | 0.14 | 0.18 | 0.69 | 0.54 | 0.07 |
| Mental health disparity | 3.76 | 3.40 | 1.12 | 1.29 | -0.30 | 3.56 | 3.67 | 1.14 | 1.21 | 0.09 |
| Feeling mentally unwell today | 4.37 | 3.91 | 1.23 | 1.46 | -0.35 | 4.05 | 4.30 | 1.33 | 1.32 | 0.19 |
| Feeling mentally unwell usually | 4.23 | 3.91 | 1.19 | 1.20 | -0.27 | 4.00 | 4.18 | 1.27 | 1.17 | 0.15 |
| Tired usually | 3.62 | 3.37 | 1.03 | 0.98 | -0.25 | 3.41 | 3.60 | 0.94 | 1.05 | 0.19 |
| Tired now | 2.51 | 2.06 | 1.53 | 1.54 | -0.29 | 2.19 | 2.44 | 1.61 | 1.51 | 0.16 |
| Tired after work | 3.01 | 2.78 | 1.06 | 1.00 | -0.23 | 2.83 | 2.99 | 1.05 | 1.04 | 0.15 |
| Feeling tired after bus travel | 2.44 | 2.45 | 1.32 | 1.31 | 0.01 | 2.25 | 2.53 | 1.17 | 1.37 | 0.22 |
| Feeling tired after train travel | 2.83 | 2.60 | 1.30 | 1.35 | -0.18 | 2.44 | 2.90 | 1.22 | 1.34 | 0.36 |

**Supplementary Table S3 – Significance of effects of COVID-19 exposure, COVID-19 course, and time since COVID-19 on health, performance, and fatigue**

|  | COVID N/Y | COVID Course | Since COVID | COVID N/Y | COVID Course | Since COVID | COVID N/Y | COVID Course | Since COVID |
| --- | --- | --- | --- | --- | --- | --- | --- | --- | --- |
| Physical sickness score | 0.231 | 0.000 | 0.124 | 0.607 | 0.000 | 0.668 | 0.198 | 0.001 | 0.037 |
| Mental sickness score | 0.958 | 0.005 | 0.674 | 0.786 | 0.003 | 0.927 | 0.746 | 0.971 | 0.582 |
| Fatigue score | 0.030 | 0.002 | 0.058 | 0.108 | 0.011 | 0.043 | 0.146 | 0.108 | 0.582 |
| Error rate score | 0.275 | 0.053 | 0.138 | 0.999 | 0.138 | 0.134 | 0.119 | 0.146 | 0.535 |
| Reaction time score | 0.436 | 0.169 | 0.401 | 0.430 | 0.062 | 0.500 | 0.838 | 0.713 | 0.668 |
|  | Source variables | | | | | | | | |
| Evolution test result | 0.355 | 0.068 | 0.120 | 0.450 | 0.135 | 0.177 | 0.034 | 0.529 | 0.186 |
| Intelligence | 0.449 | 0.724 | 0.211 | 0.027 | 0.331 | 0.043 | 0.261 | 0.696 | 0.866 |
| Cognitive reflection test | 0.031 | 0.314 | 0.271 | 0.029 | 0.363 | 0.127 | 0.654 | 0.659 | 0.715 |
| Recognition memory test | 0.846 | 0.637 | 0.414 | 0.650 | 0.793 | 0.354 | 0.857 | 0.289 | 0.567 |
| Free recall memory test | 0.672 | 0.213 | 0.513 | 0.392 | 0.268 | 0.307 | 0.579 | 0.519 | 0.898 |
| Choice test accuracy | 0.100 | 0.029 | 0.187 | NA | NA | NA | 0.081 | 0.021 | 0.174 |
| Choice test reaction time | 0.881 | 0.810 | 0.675 | 0.989 | 0.925 | 0.408 | 0.843 | 0.802 | 0.889 |
| Stroop test accuracy | 0.992 | 0.152 | 0.235 | 0.723 | 0.618 | 0.530 | 0.802 | 0.020 | 0.211 |
| Stroop test reaction time | 0.973 | 0.027 | 0.282 | 0.855 | 0.017 | 0.427 | 0.765 | 0.754 | 0.462 |
| Stroop test reaction time 1 | 0.083 | 0.003 | 0.178 | 0.028 | 0.012 | 0.325 | 0.917 | 0.220 | 0.532 |
| Stroop test reaction time 2 | 0.014 | 0.086 | 0.800 | 0.003 | 0.033 | 0.862 | 0.818 | 0.833 | 0.435 |
| Stroop test reaction time 3 | 0.663 | 0.185 | 0.582 | 0.287 | 0.089 | 0.569 | 0.535 | 0.869 | 0.949 |
| Reading time | 0.009 | 0.714 | 0.592 | 0.001 | 0.104 | 0.652 | 0.783 | 0.053 | 0.684 |
| Allergies | 0.347 | 0.022 | 0.554 | 0.626 | 0.241 | 0.966 | 0.031 | 0.012 | 0.284 |
| Skin disorders | 0.849 | 0.004 | 0.008 | 0.450 | 0.004 | 0.266 | 0.485 | 0.620 | 0.002 |
| Digestive tract disorders | 0.849 | 0.003 | 0.222 | 0.571 | 0.048 | 0.829 | 0.374 | 0.012 | 0.087 |
| Metabolic disorders | 0.016 | 0.017 | 0.998 | 0.004 | 0.424 | 0.955 | 0.619 | 0.000 | 0.945 |
| Common infectious diseases | 0.583 | 0.000 | 0.242 | 0.112 | 0.000 | 0.452 | 0.134 | 0.003 | 0.328 |
| Orthopedic disorders | 0.373 | 0.012 | 0.093 | 0.398 | 0.101 | 0.194 | 0.003 | 0.069 | 0.345 |
| Neurological disorders | 0.474 | 0.007 | 0.820 | 0.303 | 0.014 | 0.745 | 0.007 | 0.443 | 0.947 |
| Headaches | 0.078 | 0.020 | 0.630 | 0.134 | 0.080 | 0.719 | 0.439 | 0.181 | 0.167 |
| Physical pains | 0.653 | 0.063 | 0.205 | 0.816 | 0.677 | 0.988 | 0.663 | 0.018 | 0.055 |
| Chronic physical problems | 0.090 | 0.004 | 0.178 | 0.526 | 0.060 | 0.662 | 0.018 | 0.022 | 0.065 |
| Antibiotics in the last year | 0.456 | 0.048 | 0.776 | 0.674 | 0.025 | 0.845 | 0.474 | 0.888 | 0.721 |
| Antibiotics in the last 3 years | 0.202 | 0.043 | 0.452 | 0.285 | 0.007 | 0.433 | 0.544 | 0.501 | 0.678 |
| Doctor visits | 0.106 | 0.001 | 0.998 | 0.127 | 0.000 | 0.716 | 0.285 | 0.531 | 0.633 |
| Hospital visits in the past 5 years | 0.117 | 0.013 | 0.234 | 0.015 | 0.052 | 0.239 | 0.251 | 0.066 | 0.812 |
| Prescribed drugs for physical health | 0.572 | 0.592 | 0.961 | 0.135 | 0.755 | 0.538 | 0.262 | 0.682 | 0.279 |
| Physical health disparity | 0.017 | 0.076 | 0.487 | 0.000 | 0.807 | 0.826 | 0.828 | 0.023 | 0.385 |
| Feeling physically unwell today | 0.085 | 0.004 | 0.749 | 0.725 | 0.111 | 0.243 | 0.012 | 0.020 | 0.324 |
| Feeling physically unwell usually | 0.371 | 0.008 | 0.953 | 0.297 | 0.071 | 0.692 | 0.863 | 0.062 | 0.569 |
| Expected shorter lifespan | 0.983 | 0.129 | 0.556 | 0.215 | 0.732 | 0.311 | 0.125 | 0.025 | 0.940 |
| Depression | 0.969 | 0.022 | 0.664 | 0.915 | 0.022 | 0.901 | 0.860 | 0.943 | 0.432 |
| Anxiety | 0.062 | 0.000 | 0.729 | 0.131 | 0.000 | 0.472 | 0.160 | 0.987 | 0.784 |
| Phobia | 0.507 | 0.433 | 0.516 | 0.328 | 0.935 | 0.411 | 0.806 | 0.107 | 0.734 |
| Obsession | 0.599 | 0.639 | 0.015 | 0.455 | 0.543 | 0.024 | 0.993 | 0.768 | 0.378 |
| Other mental health problems | 0.116 | 0.000 | 0.654 | 0.242 | 0.000 | 0.483 | 0.238 | 0.922 | 0.504 |
| Prescribed drugs for mental health | 0.187 | 0.015 | 0.399 | 0.084 | 0.030 | 0.217 | 0.665 | 0.320 | 0.298 |
| Mental health disparity | 0.474 | 0.001 | 0.745 | 0.560 | 0.002 | 0.993 | 0.873 | 0.278 | 0.736 |
| Feeling mentally unwell today | 0.280 | 0.001 | 0.305 | 0.797 | 0.002 | 0.520 | 0.048 | 0.338 | 0.387 |
| Feeling mentally unwell usually | 0.094 | 0.087 | 0.547 | 0.389 | 0.028 | 0.786 | 0.156 | 0.579 | 0.488 |
| Tired usually | 0.031 | 0.001 | 0.255 | 0.069 | 0.009 | 0.421 | 0.291 | 0.054 | 0.397 |
| Tired now | 0.159 | 0.000 | 0.348 | 0.653 | 0.000 | 0.434 | 0.047 | 0.298 | 0.711 |
| Tired after work | 0.084 | 0.113 | 0.042 | 0.050 | 0.398 | 0.036 | 0.837 | 0.114 | 0.653 |
| Feeling tired after bus travel | 0.085 | 0.060 | 0.248 | 0.269 | 0.235 | 0.130 | 0.209 | 0.122 | 0.883 |
| Feeling tired after train travel | 0.001 | 0.046 | 0.074 | 0.070 | 0.056 | 0.049 | 0.004 | 0.503 | 0.727 |

*The table shows the results (p-values) of two-sided tests. The significances lower than 0.0005 are printed as 0.000.*

**Supplementary Figure S1 – Third-degree polynomial trajectories of the disease course for health, performance, and fatigue over a span of three years**


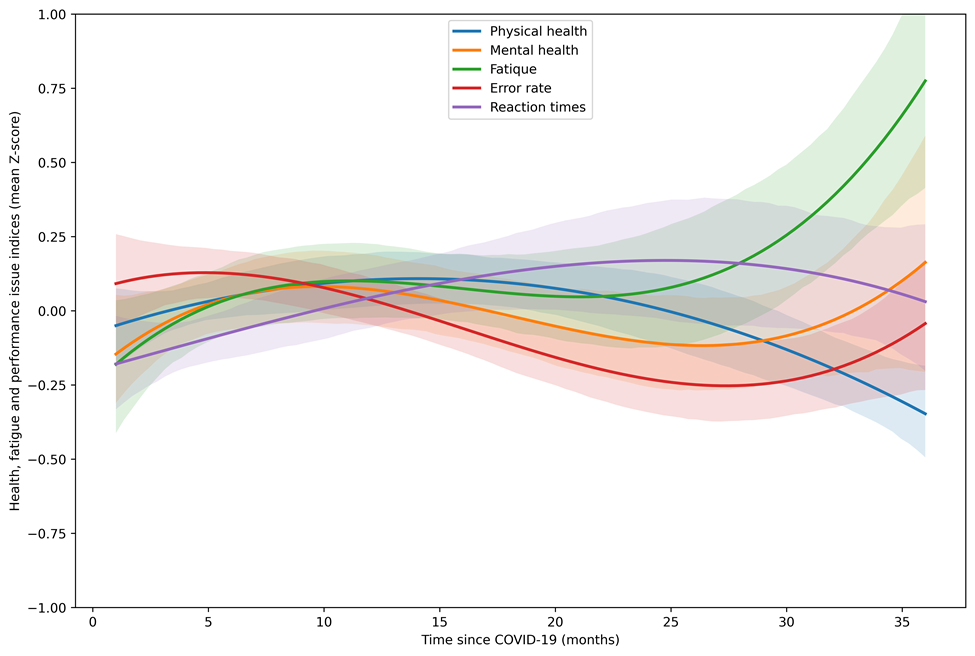


*This scatterplot illustrates the relationships between five health-related variables (Physical Health, Mental Health, Fatigue, Error Rate, and Reaction Times) and the time elapsed since contracting COVID-19. Each variable is represented by a unique color, with data points fitted by a third-degree polynomial curve to visualize the trends. The bands around the lines represent 80% Confidence Intervals.*
